# Validation of the 12‐item World Health Organization Disability Assessment Schedule 2.0 in individuals with schizophrenia, depression, anxiety, and diabetes in Singapore

**DOI:** 10.1371/journal.pone.0294908

**Published:** 2023-11-30

**Authors:** Edimansyah Abdin, Vanessa Seet, Anitha Jeyagurunathan, Sing Chik Tan, Yee Ming Mok, Swapna Verma, Eng Sing Lee, Mythily Subramaniam

**Affiliations:** 1 Research Division, Institute of Mental Health, Singapore, Singapore; 2 Department of Mood and Anxiety, Institute of Mental Health, Singapore, Singapore; 3 Department of Psychosis, Institute of Mental Health, Singapore, Singapore; 4 Clinical Research Unit, National Healthcare Group Polyclinics, Singapore, Singapore; 5 Saw Swee Hock School of Public Health, National University of Singapore, Singapore, Singapore; University of Perugia: Universita degli Studi di Perugia, ITALY

## Abstract

**Background:**

There is limited evidence on the reliability and validity of the 12-item World Health Organization Disability Assessment Schedule 2.0 (WHODAS 2.0) in an Asian patient population with mental and physical disorders. The current study aimed to examine the psychometric properties of the WHODAS 2.0 among patients with schizophrenia, depression, anxiety, and diabetes.

**Methods:**

A total of 1076 patients (M  =  40.9 years, SD  =  14.7) were recruited from the outpatient clinics of a tertiary psychiatric hospital and a primary care clinic. Internal consistency and test-retest reliability, structural validity, convergent validity, agreement, and floor and ceiling effects were examined.

**Results:**

Our confirmatory factor analysis (CFA) showed that the 1-factor model fits our data. Multigroup CFA demonstrated metric and scalar invariance, indicating the scores can be compared across the four conditions. The WHODAS 2.0 scale had excellent reliability in the overall sample and good to excellent reliability across conditions. The test-retest reliability and agreement between self-administered and interviewer-administered modes were good. The WHODAS 2.0 scores had moderate to strong correlations with the Social and Occupational Functioning Scale and the Sheehan Disability Scale scores in the overall sample and across four conditions.

**Conclusion:**

Findings suggest that the WHODAS 2.0 is a valid tool to measure functioning and disability in those with schizophrenia, anxiety, depression, and diabetes in an Asian patient population.

## Introduction

A growing number of studies have employed the World Health Organization Disability Assessment Schedule 2.0 (WHODAS 2.0) to assess disability [1–3]. The WHODAS 2.0 is a generic instrument developed by the World Health Organization to provide a standardized assessment tool for disability across diseases and cultures [1]. The instrument has demonstrated good face validity, including replicability across countries, population groups, diagnostic groups, ages, and genders [2]. The WHODAS 2.0 has three versions (a 36-item, 12-item, and 12 + 24-item version). The 12-item version has been derived from the 36-item version as a shorter version for assessing overall functioning in surveys or health outcome studies [3]. The 12-item version is short, simple, easy to administer in both clinical and general population settings and takes about 5 to 20 minutes to complete. The 12-item version is preferred over the 36-item version and reported to have good internal consistency, discriminant validity, construct validity [4–6], and considered more responsive than the 36-item version [7].

Previous studies have examined the psychometric properties of the 12-item version of the WHODAS 2.0 among patients with anxiety and stress disorders [4], musculoskeletal pain conditions [8, 9], neurosurgical patients [10], major depressive disorder [3], schizophrenia and psychotic disorders [5, 11], trauma [6], autism spectrum [12], Huntington disease [13, 14], Chagas disease [15], Parkinson disease [16], postpartum women with and without severe maternal morbidity [17] and in children, young, and older adults populations [8, 18–23]. Overall, the studies found that the instrument has high internal consistency, convergent validity, and adequate structural validity across various hypothetical models, including a 1-factor model with and without correlated residuals, a 6-factor model, and a single second-order with 6 first-order factor model. The internal consistency of the instrument as measured by the Cronbach’s alpha was high and ranged from 0.83 to 0.94 [3, 4, 12, 14]. Correlation with other measures such as EuroQol-5D (EQ-5D) utility and RAND-12 Health Status Inventory’s physical health and mental health scores has been established and ranged from -0.41 to -0.76 [14]. Additionally, the correlation with Montgomery-Åsberg Depression Rating Scale-self-report version (MADRS-S), Generalized Anxiety Disorder 7-item scale (GAD-7), and Sheehan Disability Scale (SDS) ranged from 0.58 to 0.66, respectively [4]. A systematic review of 14 studies among a general adult population or people with acute physical causes of disability found that the WHODAS 2.0 was reliable, had a good correlation with other disability measures and was multidimensional [8].

Despite an increasing number of studies examining the psychometric properties of the WHODAS 2.0, little is known about its psychometric performance in Asian patient populations. There is a gap in our knowledge about the performance of this instrument across mental and physical health problems in the Singapore context, especially among patients with mental disorders such as schizophrenia, depression, anxiety, and chronic physical conditions such as diabetes. Singapore is a highly urbanized country in South-East Asia with a resident population of 4 million, consisting of 74.1% Chinese, 13.6% Malays, 9% Indians, and 3.3% of those belonging to other ethnicities. The resident population has a high literacy rate of 97.1% and is mostly (70.5%) literate in more than one language. English is the primary language of most of the resident population. In Singapore, the prevalence of major depressive disorder (MDD), and generalized anxiety disorder (GAD) showed an increasing trend over the years. The Singapore Mental Health Study (SMHS) reported that the prevalence of MDD (6.2% versus 5.6%) and GAD (1.79% versus 0.9%) [24] were higher in 2016 than in 2010 [25]. Furthermore, both men and women with mental disorders had higher disability than those without mental disorders [26]. Apart from these conditions, schizophrenia and diabetes are two of the most serious mental and physical health conditions in Singapore. The conditions are significant to both the public and policy makers due to their associated complications, and significant impact on the patients, families, health systems and national economy [27, 28]. Schizophrenia has a relatively low lifetime prevalence (2.3%) in the population, and it has been suggested that the severe symptoms and associated disability underlie the need for ongoing outreach and treatment [27]. Meanwhile, data from the National Population Health Survey has suggested that the overall crude prevalence of diabetes showed an increasing trend from 2007 (4.9%) to 2021 (6.9%) [29]. In 2016, Singapore’s minister of health declared a “War on Diabetes” to rally the entire nation to tackle diabetes. Furthermore, diabetes is a common comorbidity in patients with mental health conditions in Singapore [30–32]. Thus, it is essential to establish the validity of the WHODAS 2.0 as a generic functioning and disability measure that can be adopted across programmes. This further ensures that the assessment of functioning and disability among mental health patients who seek treatment for their comorbid conditions is based on a similar measure.

There are few studies examining the reliability and validity of the WHODAS 2.0 in the Asian population and assessing the comparability of this instrument to disease-specific disability measures in the clinical setting. Disability has increasingly become an important indicator of disease burden and for evaluating the effectiveness of health interventions [33–35]. With the significant increase in healthcare treatment and community mental health care programmes across the country, there is an urgent need to evaluate the effectiveness of these programmes using reliable and accurate measures of patient’s functioning and disability that are comparable not only across health conditions, but also across treatments and programmes of care. Hence, the main objective of the present study is to examine the psychometric properties of the WHODAS 2.0 among patients with common mental and physical disorders including schizophrenia, depression, anxiety, and diabetes. More specifically, we analyzed the instrument’s structural validity, internal consistency, agreement, and convergent validity in patients with the specific conditions.

## Methods

The study was approved by the Singapore’s NHG DOMAIN SPECIFIC REVIEW BOARD (DSRB). NHG DSRB Reference Number: 2018/01143. A cross-sectional study was conducted on a convenience sample from June 2019 to November 2022 at the Institute of Mental Health (IMH), National Healthcare Group Polyclinics (NHGP), and the Community Wellness Clinics (CWC) in Singapore. Briefly, IMH is a tertiary psychiatric hospital in Singapore. NHGP is a government-funded primary care center that includes a one-stop center for patients with diabetes. Meanwhile, CWC is a community clinic that provides comprehensive and integrated care for patients including those with mental disorders in Singapore. Participants were included in this study if they were diagnosed by psychiatrists/ primary care physicians with schizophrenia spectrum disorders, depression, anxiety disorders, or diabetes, Singapore citizens and Permanent Residents (PRs), fluent in English, and aged 21 years and over. Participants were excluded if they were non-Singapore citizens and non-PRs, unable to speak and read in English, less than 21 years of age and incapable of doing an interview due to severe physical or mental conditions. Patients with depression, anxiety, and schizophrenia were recruited from IMH and CWC, while diabetes patients were recruited from the NHGP. The study also recruited patients for test-retest (at two weeks) assessment to establish test-retest reliability using a convenience sampling among the patients who agreed to participate in the main study. Patient’s family members, other relatives or caregivers were recruited as proxy assessors to establish agreement between mode of administration i.e., self-administered,. proxy-administered, and rater-administered (assessed by our trained staff) on selected respondents to establish agreement between self-administered and rater-administered scores. Written informed consent was obtained from all study participants.

### Instruments

The self-administered English version of the WHODAS 2.0 questionnaire contains 12 items covering individual domains of functioning based on the ICF. The questionnaire assesses the disability during the preceding 30 days. Each item uses a 5-point Likert-type scale to reflect the level of difficulty, starting with ‘no difficulty’ and increasing in an ordered fashion from ‘mild’, ‘moderate’, ‘severe’ to ‘extreme or cannot do’. A simple scoring can be generated by assigning each of the items a score ranging from 0 (mild) -4 (extreme or cannot do)–which are then summed up with total scores ranging from 0 to 48. The total score can be converted to a 0–100 scale by the sum of all answers divided by 48 and multiplied by 100 where a score of 0% represents the highest possible level of functioning and independence, while a score of 100% represents the lowest level of functioning with total dependence.

Data on the following instruments were also collected from the respondents. The English version of the Social and Occupational Functioning Scale (SOFAS) [36], which is an interviewer-rated scale was used to measure social and occupational functioning in all patients. The SOFAS total scores range from 0 to 100, with higher scores denoting better functioning. The English version of the Sheehan Disability Scale (SDS) [37] is a self-administered and generic scale to measure functional impairment in three areas: work/school, social life, and home/family responsibilities. The total scores can be generated by averaging the three items, with higher scores denoting poor functional impairments. In this study, the English version of the WHODAS 2.0 and SDS were self-administered by the respondents, while the English version of the SOFAS were administered by the interviewers.

Socio-demographic information including age, gender, education, ethnicity, marital and employment status, as well as psychiatric diagnosis were also collected from the participants.

### Sample size

The sample size was calculated using a semPower [38] based on a 1-factor model of the WHODAS 2.0 with 54 degrees of freedom (df) found in a prior study [19]. A power of 80% and an alpha error level of 0.05 were utilized. A minimum sample size of at least 233 subjects was needed for specific-conditions, to ensure the confirmatory factor analysis (CFA) estimation with adequate power to obtain an acceptable cut-off of misfit model, corresponding to a Root Mean Square Error Approximation (RMSEA) of at least 0.05.

### Statistical analysis

All analyses were performed in Stata version 15.1 (Stata Corp, USA) and RStudio software version 2022.07.2. Ceiling and floor effects were calculated as the percentage of participants who achieved the maximum and minimum scores for each item. We adopted the standard approach for assessing the psychometric properties of the measures. This includes internal consistency, structural validity, and, convergent validity. Agreement was assessed using the intraclass correlation coefficient (ICC) based on a two-way mixed model with absolute agreement. ICC was interpreted using the following criteria [39]: >0.9, excellent reliability, 0.75 to 0.9, good, 0.5 to 0.75, moderate, and <0.5, poor reliability. Internal consistency was examined using Cronbach alpha [40], complemented by composite reliability index [41]. Cronbach alpha ≥0.7 is usually regarded as acceptable [40]. The validity of factor structure models proposed by previous studies was examined through confirmatory factor analysis (CFA) using diagonal weighted least squares estimator. The following models were tested including the 1-factor model (Model 1) [6, 10, 20], followed by a 1-factor model with 1 correlated residual (Model 2) [20], a 1-factor model with 3 correlated residuals (Model 3) [3], a 6-factor model (Model 4) [14, 23], and a second-order with 6 first-order factors model (Model 5) [5, 12, 42]. However, the covariance matrices of models 4 and 5 were not positive definite and had negative error variances. The estimates were not reported because the model failed to converge. The model fit was assessed using the three indices including root mean square error of approximation (RMSEA), comparative fit index (CFI), and Tucker-Lewis index (TLI). The CFI values above 0.95 and TLI values above 0.90 are considered to be of excellent fit [43], while RMSEA values below 0.8 are considered to be acceptable [44]. The overall model fit was considered an adequate fit if at least two of these three indices met their respective cut-off [45–47]. We also tested measurement invariance of the WHODAS 2.0 across the four conditions through multiple-group CFA (MGCFA). The MGCFA first performed CFA independently in each subgroup to establish the appropriateness of a baseline model. Subsequently, the MGCFA was conducted to establish measurement invariance across subgroups at each level of the three measurement invariance models including (1) configural model (i.e., separates the sample into four subgroups, but no parameter constraints are imposed); (2) metric model (i.e., constrains the factor loadings to be equal across subgroups); and (3) scalar model (i.e., constrains the factor loadings and the item intercepts to be equal across subgroups) [48]. Each measurement invariance model was considered established if two or more changes in the following criteria were satisfied: change in the chi-squared values (Δχ2) indicated a p-value >0.05, ΔCFI ≥ −0.010, ΔTLI ≥ −0.010 or ΔRMSEA ≤ 0.015 [49–51]. The convergence between the WHODAS 2.0 and other measures was examined using Spearman’s rho (r) correlation coefficients due to the skewed distribution of the data. We used the following categories for evidence of convergent validity: >0.6, very strong; ≥0.5 to <0.6, strong; <0.5 to ≥ 0.3, moderate; and <0.3, weak [52].

## Results

### Sample characteristics

Sample characteristics are presented in Table 1. A total of 1076 patients were recruited for the study. The sample comprised 48.0% female and 52.0% male respondents. The mean age of the overall sample was 40.9 years (SD = 14.7; range = 21–82 years), 67.1% were Chinese, 13.9% were Malays, 14.5% were Indians, and 4.5% belonged to other ethnicities. Of the respondents, 25.9% (n = 279) had schizophrenia, 25.8% (n = 278) had depression, 22.4% (n = 241) had anxiety and 25.8% (n = 278) had diabetes.

**Table 1:** Sociodemographic characteristics (N = 1076).

	Mean	SD
Age	40.9	14.7
	n	%
Gender		
Male	560	52.0
Female	516	48.0
Ethnicity		
Chinese	722	67.1
Malay	150	13.9
Indian	156	14.5
Others	48	4.5
Education		
Primary	47	4.4
Secondary	260	24.2
Pre-U/Junior college	46	4.3
Vocational Institute /ITE	126	11.7
Diploma	304	28.3
University	291	27.1
Marital status		
Never married	607	56.8
Currently married	338	31.6
Separated	12	1.1
Divorced	96	9.0
Widowed	15	1.5
Diagnosis		
Schizophrenia	279	25.9
	Mean	SD
PANSS total scores	46.2	13.0
	n	%
Depression	278	25.8
None/Minimal	38	13.7
Mild	69	24.9
Moderate	72	25.9
Moderately severe	63	22.7
Severe	36	13.0
Anxiety	241	22.4
None/Minimal	46	19.1
Mild	69	28.6
Moderate	53	22.0
Severe	73	30.3
Diabetes	278	25.8
HbA1c < 6.5	45	17.05
HbA1c ≥ 6.5	219	82.95

### Confirmatory factor analysis

A series of hypothetical models were tested using CFA to examine the factor structure of the WHODAS 2.0 shown in Table 2. According to the goodness-of-fit indices criteria, Model 3 with three error covariances was chosen as the best fitting model. The goodness-of-fit indices were acceptable (X^2^ = 309.417, df = 51, CFI = 0.994, TLI = 0.994, RMSEA = 0.069) (Table 2). The factor loadings were all significant (P value < 0.001) and ranged from a minimum of 0.64 to a maximum of 0.86.

**Table 2:** Confirmatory factor analysis (CFA) results.

Model		*x* ^2^	*df* ^2^	CFI	TLI	RMSEA
Model 1	1-factor^a^	1115.176	54	0.981	0.977	0.136
Model 2	1-factor, covariance e1~e7	948.887	53	0.984	0.980	0.126
Model 3	1-factor, covariance e1~e7, e8~e9, e10~e11	309.417	51	0.994	0.994	0.069

### Measurement invariance

Results of measurement invariance tests of the WHODAS 2.0 using MGCFA across the four conditions are shown in Table 3. The factor structure of the WHODAS 2.0 had adequate fit to the data for each subgroup (schizophrenia: RMSEA = 0. 058, CFI = 0.996, TLI = 0.995; depression: RMSEA = 0. 074, CFI = 0.993, TLI = 0.991; anxiety: RMSEA = 0.057, CFI = 0.996, TLI = 0.995; diabetes: RMSEA = 0. 061, CFI = 0.997, TLI = 0.996). This suggests that the model meets the criteria for configural measurement invariance tests across subgroups. In the configural model, which tests whether the same pattern of factor structure is present across each subgroup, the model demonstrated adequate fit across subgroups (RMSEA = 0. 063, CFI = 0.996, TLI = 0.995). Next, the metric model was tested in which the factor loadings were constrained to be equal across subgroups. The change in the three fit indices between configural and metric model suggested that the fit of the metric model was satisfied (ΔCFI = −0.002, ΔTLI = −0.002, ΔRMSEA = 0.009). Hence, the metric measurement invariance was established. Subsequently, scalar model was tested in which factor loadings and thresholds were constrained to be equal across subgroups. The change in the three fit indices between metric and scalar models suggests that the fit of the scalar model was satisfied (ΔCFI = −0.004, ΔTLI = −0.002, ΔRMSEA = 0.007). This suggests that the scalar measurement invariance model of the WHODAS 2.0 was supported in this study.

**Table 3:** Tests of measurement invariance.

	*x* ^2^	*df*	CFI	TLI	RMSEA	Δχ2(*df*)	P value	ΔCFI	ΔTLI	ΔRMSEA
Baseline										
Schizophrenia	99.046	51	0.996	0.995	0.058					
Depression	126.945	51	0.993	0.991	0.074					
Anxiety	90.973	51	0.996	0.995	0.057					
Diabetes	102.946	51	0.997	0.996	0.061					
Configural: No constraints	421.231	204	0.996	0.995	0.063					
Metric: Equal factor loadings	566.452	237	0.994	0.993	0.072	145.22(33)	<0.001	-0,002	-0.002	0.009
Scalar: Equal factor loadings and thresholds	859.503	321	0.990	0.991	0.079	293.05(84)	<0.001	-0.004	-0.002	0.007

### Item score distribution, floor, and ceiling effects

Descriptive statistics of the items are presented in Table 4. Generally, patients rated high mean scores on items concerning how emotionally affected they were by their health problem, difficulties participating in society and everyday life activities (S5, S4, S12), and lower mean scores on items that concerned functional impairment in self-care such as getting dressed and washing their whole body (S8, S9). The distribution of these two items (S8, S9) exhibits high skewness and kurtosis. There was also evidence of floor effects regarding several items. The floor effect (an answer of “no problem”) value ranged from 25.6% (for item 5) to 78.6% (for item 8), while the ceiling effect (an answer of “extremely large” or “cannot do”) ranged from 0.4% (for item 9) to 3.8% (for item 12).

**Table 4:** Descriptive statistics of the items.

	Overall sample				Schizophrenia		Depression		Anxiety		Diabetes	
	mean	SD	Ceiling	Floor	Mean	SD	Mean	SD	Mean	SD	Mean	SD
S1. Standing for long periods such as 30 minutes	0.71	1.01	1.40	59.46	0.81	1.04	0.79	1.04	0.70	1.03	0.55	0.88
S2. Taking care of your household responsibilities	0.94	1.07	2.33	46.74	0.95	1.06	1.26	1.10	1.05	1.11	0.53	0.85
S3. Learnings a new task, for example, learning how to get to a new place	0.78	0.99	1.31	53.26	0.93	1.06	0.99	1.05	0.85	0.95	0.36	0.72
S4. How much of a problem did you have joining in community activities	1.02	1.18	3.82	47.16	0.95	1.09	1.44	1.25	1.30	1.24	0.44	0.82
S5. How much have you been emotionally affected by your health problem	1.47	1.17	3.63	25.61	1.23	1.09	2.07	1.07	1.90	1.10	0.73	0.86
S6. Concentrating on doing something for ten minutes	0.89	1.02	1.21	48.00	0.86	1.00	1.31	1.03	1.14	1.07	0.29	0.65
S7. Walking a long distance such as a kilometre	0.79	1.03	1.77	54.70	0.87	1.05	0.83	1.04	0.78	1.06	0.67	0.98
S8. Washing your whole body	0.38	0.80	0.65	77.67	0.43	0.83	0.42	0.81	0.42	0.88	0.25	0.66
S9. Getting dressed	0.34	0.74	0.37	78.58	0.39	0.78	0.43	0.80	0.37	0.77	0.19	0.57
S10. Dealing with people you do not know	0.90	1.09	2.42	49.58	0.92	1.07	1.25	1.13	1.19	1.19	0.29	0.64
S11. Maintaining a friendship	0.91	1.11	2.70	51.49	0.92	1.10	1.28	1.15	1.16	1.19	0.29	0.68
S12. Your day-to-day work	1.02	1.17	3.82	46.74	1.04	1.15	1.44	1.22	1.32	1.21	0.34	0.71
Cronbach’s alpha		0.92				0.91		0.89		0.92		0.92
Composite reliability		0.94				0.94		0.92		0.94		0.96

### Reliability

Internal reliability showed that the WHODAS 2.0 scale had excellent reliability in the overall sample and good to excellent reliability across subgroups. The Cronbach’s alpha of the WHODAS 2.0 was 0.92. The composite reliability was 0.94. The scale had the highest reliability in the diabetes subsample, with Cronbach’s alpha and composite reliability values of 0.92 and 0.96, respectively (Table 4). The agreement between self-administered and interviewer-administered mode, estimated based on an ICC was good (0.89, 95% CI: 0.78 to 0.95). The test-retest reliability coefficient was also good (ICC = 0.78, 95% CI: 0.58 to 0.89) while the agreement between self-administered and proxy-administered mode was considered poor reliability (ICC = 0.40, 95% CI: 0.03 to 0.67) (S1 Table).

### Convergent validity

The correlation coefficient between the WHODAS 2.0 and the SOFAS global rating of current functioning, was strong in the overall sample (r = -0.57) and moderate to strong across the four disorders (schizophrenia, r = -0.38, depression, r = -0.51, anxiety, r = -0.59 and diabetes, r = -0.42). The correlation coefficient between the WHODAS 2.0 and the SDS total score was very strong in the overall sample (r = 0.77) and across the four disorders (schizophrenia, r = 0.68, depression. r = 0.70, anxiety, r = 0.80 and diabetes, r = 0.61) (S2 Table).

## Discussion

In order to provide evidence of the validity and reliability of the WHODAS 2.0 as a useful tool to measure functioning and disability in those with schizophrenia, anxiety, depression, and diabetes in a multi-ethnic Asian population in Singapore, this study examined the factor structure, measurement invariance, internal consistency reliability, agreement, and convergent validity of the scale across these four conditions. In our confirmatory factor analysis, the goodness-of-fit results (X^2^ = 309.417, df = 51, CFI = 0.994, TLI = 0.994, RMSEA = 0.069) confirmed that the 1-factor model fits our data. Similar results were also found among patients with first major depressive episode and a pooled dataset from adult community samples [3, 20]. We have also examined a 6-factor model and second-higher order with six first-order factor model in this sample; however, we found that extending beyond a one-factor model yields unreliable results because of a convergence issue. Based on these findings, we suggest that the WHODAS 2.0 is best described by a 1-factor model. In addition, this study also found that the goodness-of-fit statistics for RMSEA, CFI and TLI were good within each sample of patients with schizophrenia, anxiety, depression, and diabetes. Further investigation on measurement invariance in terms of configural, metric and scalar invariance across these four conditions found the MGCFA results strongly support the measurement invariance of the WHODAS 2.0, indicating that the 1-factor structure model is appropriate across the conditions. These findings have practical implications for users who are interested in using the WHODAS 2.0 as the results seem to support the use of total scores for examining disability within groups and undertaking mean group comparisons between these four conditions. For example, clinicians and researchers will be able to evaluate the effectiveness of relevant programs or interventions using WHODAS 2.0 as a reliable and accurate measure that can capture all aspects of patient functioning and disability that is comparable across health conditions. Moreover, assessing disability across health conditions is important for identifying needs when planning healthcare services, setting priorities, allocating resources and evaluating outcomes and effectiveness of interventions [53].

In terms of reliability, our findings consistently showed that the WHODAS 2.0 internal consistency was reliable in the overall sample and across conditions. The internal consistency of the WHODAS 2.0 in those with schizophrenia, depression, anxiety, and diabetes (Cronbach’s alpha = 0.88–0.91) were higher than 0.70 as recommended by Kline (1979). These findings are comparable to the previous studies conducted among patients with anxiety and stress disorders (Cronbach’s alpha = 0.83–0.92) [4], autism spectrum disorder (Cronbach’s alpha = 0.86) [12], Huntington disease (Cronbach’s alpha = 0.94) [14], and first major depressive episode (Cronbach’s alpha = 0.89) [3]. We found mixed evidence of test-retest reliability and agreement between self-administered, interviewer-administered, and proxy-administered of the WHODAS 2.0 in our sample. The analysis found that the WHODAS 2.0 test-retest reliability was good (ICC = 0.78), indicating the instrument’s ability to produce reproducible results when used repeatedly over a short period of time. This finding appears to be in line with earlier studies, which found the test–retest reliability for the WHODAS 2.0 was in the good range, with ICC of 0.70 or higher in patients with anxiety and stress (ICC = 0.83) [4] and Persian traffic cohort sample (ICC = 0.97) [54]. We also found that the WHODAS 2.0 agreement between self-administered and interviewer-administered modes was good (ICC = 0.89), indicating that functioning and disability ratings by patients and interviewers were generally consistent and therefore considered reliable. However, in considering proxy assessment for the WHODAS 2.0, our study found that the agreement between proxy-administered and self-administered mode was poor (ICC = 0.40) and less reliable than the agreement between self-administered and interviewer-administered modes. This finding is not in line with a previous study among stroke rehabilitation patients, which reported that agreement between self-administered and proxy-administered versions were good (ICC = 0.799) [55]. Our finding seems to suggest that patients’ experiences or interpretations of their functioning or impairment may differ from those reported by their proxies.

In this study we found the WHODAS 2.0 has good convergent validity with other disability measures. The WHODAS 2.0 scores had moderate to strong correlations with SOFAS and SDS scores in the overall sample and across four conditions. These findings are in line with the earlier studies that showed a significant correlation between WHODAS 2.0 and SOFAS among people with mental disorders without psychotic symptoms (r = -0.245) [56]. Similarly, a strong correlation between WHODAS 2.0 and SDS among patients with anxiety and stress (r = 66) has also been observed [4]. Hence, these results suggest that the WHODAS 2.0 is psychometrically valid for use in our sample with good convergence between the SOFAS and SDS.

Our study has some limitations. Given that we only included patients with schizophrenia, anxiety, depression and diabetes, the generalizability of our findings to other mental and physical conditions needs further investigation. Another limitation of this study is that the measures of the current study were administered only in English. Hence, the validity and reliability among those who were not fluent in English remain uncertain. However, given that English is the main language of Singaporean residents, we believe the current findings are useful for majority of our patient’s population in Singapore. Another limitation is that we didn’t examine how well the individual items fitted the Item Response Theory (IRT) models. A previous study has shown the level of disability especially in item related to “participation in the society domain” was high in psychiatric outpatient’s sample [57], which needs to be further explored in future studies. Despite the limitations, to our knowledge, this is the first study in a multi-ethnic Asian population that directly examines the validity and reliability of the WHODAS 2.0 in patients with schizophrenia, anxiety, depression and diabetes and provides important evidence about the usefulness of the tool for assessing disability across mental and physical problems in Singapore.

## Supporting information

S1 Checklist STROBE statement—Checklist of items that should be included in reports of observational studies. (DOCX)

S1 Table Intraclass correlation coefficient (ICC) of the WHODAS 2.0. (DOCX)

S2 Table Correlation coefficients between the WHODAS 2.0, SOFAS and SDS. (DOCX)
