# Supplementary material for: Validation of the 12‐item World Health Organization Disability Assessment Schedule 2.0 in individuals with schizophrenia, depression, anxiety, and diabetes in Singapore
Source: PLoS One. 2023 Nov 30;18(11):e0294908. doi: 10.1371/journal.pone.0294908 (PMC10688897; doi:10.1371/journal.pone.0294908)
Supplement: S1 Table — (DOCX) [file pone.0294908.s002.docx]

Supplementary Table 5. Intraclass correlation coefficient (ICC) of the WHODAS 2.0

| Reliability/Agreement | N | ICC | 95% CI |
| --- | --- | --- | --- |
| Test-retest reliability | 30 | 0.78 | 0.584-0.89 |
| Agreement |  |  |  |
| Self-administered vs. Interviewer-administered | 30 | 0.89 | 0.78-0.95 |
| Self-administered vs. Proxy-administered | 30 | 0.40 | 0.03-0.67 |

ICC values > 0.9 is usually regarded as excellent, 0.75-0.90 as good, 0.50 0.74 as poor [30].
