# Supplementary material for: Validation of the 12‐item World Health Organization Disability Assessment Schedule 2.0 in individuals with schizophrenia, depression, anxiety, and diabetes in Singapore
Source: PLoS One. 2023 Nov 30;18(11):e0294908. doi: 10.1371/journal.pone.0294908 (PMC10688897; doi:10.1371/journal.pone.0294908)
Supplement: S2 Table — (DOCX) [file pone.0294908.s003.docx]

Supplementary Table 6. Correlation coefficients between the WHODAS 2.0, SOFAS and SDS

|  | WHODAS 2.0 |  |
| --- | --- | --- |
|  | Spearman’s r | p value |
| SOFAS |  |  |
| Overall sample | -0.568 | <0.001 |
| Schizophrenia | -0.377 | <0.001 |
| Depression | -0.513 | <0.001 |
| Anxiety | -0.590 | <0.001 |
| Diabetes | -0.415 | <0.001 |
| SDS |  |  |
| Overall sample | 0.766 | <0.001 |
| Schizophrenia | 0.677 | <0.001 |
| Depression | 0.696 | <0.001 |
| Anxiety | 0.802 | <0.001 |
| Diabetes | 0.610 | <0.001 |
